# Effectiveness of a ‘do not interrupt’ vest intervention to reduce medication errors during medication administration: a multicenter cluster randomized controlled trial

**DOI:** 10.1186/s12912-021-00671-7

**Published:** 2021-08-24

**Authors:** Sarah Berdot, Aurélie Vilfaillot, Yvonnick Bezie, Germain Perrin, Marion Berge, Jennifer Corny, Thuy Tan Phan Thi, Mathieu Depoisson, Claudine Guihaire, Nathalie Valin, Claudine Decelle, Alexandre Karras, Pierre Durieux, Laetitia Minh Maï Lê, Brigitte Sabatier

**Affiliations:** 1 Pharmacy Department, Hôpital européen Georges-Pompidou, APHP, 20 rue Leblanc, 75015 Paris, France; 2 INSERM, UMRS1138, Centre de Recherche des Cordeliers, Sorbonne Université, Université de Paris, Paris, France; 3 Clinical Research Department, Hôpital européen Georges-Pompidou, APHP, 20 rue Leblanc, 75015 Paris, France; 4 Pharmacy Department, Paris Saint Joseph Hôpital, Paris, France; 5 Pharmacy Department, Hôpital Vaugirard and Hôpital Corentin Celton, APHP, Paris, France; 6 DSAP, Hôpital européen Georges-Pompidou, APHP, 20 rue Leblanc, 75015, Paris, France; 7 Department of Nephrology, Hôpital européen Georges-Pompidou, APHP, 20 rue Leblanc, 75015, Paris, France; 8 Paris Descartes University, Paris, France; 9 INSERM, PARCC, Paris, France; 10 Lip(Sys)2, EA7357, UFR Pharmacie, U-Psud, University of Paris-Saclay, Paris, France

**Keywords:** Interruptions, Vest, Medication errors/nursing* (MeSH), Medication errors/prevention and control (MeSH), Nursing staff, Hospital / organization & administration (MeSH), Safety management / organization & administration* (MeSH)

## Abstract

**Background:**

The use of a ‘do not interrupt’ vest during medication administration rounds is recommended but there have been no controlled randomized studies to evaluate its impact on reducing administration errors. We aimed to evaluate the impact of wearing such a vest on reducing such errors. The secondary objectives were to evaluate the types and potential clinical impact of errors, the association between errors and several risk factors (such as interruptions), and nurses’ experiences.

**Methods:**

This was a multicenter, cluster, controlled, randomized study (March–July 2017) in 29 adult units (4 hospitals). Data were collected by direct observation by trained observers. All nurses from selected units were informed. A ‘Do not interrupt’ vest was implemented in all units of the experimental group. A poster was placed at the entrance of these units to inform patients and relatives. The main outcome was the administration error rate (number of Opportunities for Error (OE), calculated as one or more errors divided by the Total Opportunities for Error (TOE) and multiplied by 100).

**Results:**

We enrolled 178 nurses and 1346 patients during 383 medication rounds in 14 units in the experimental group and 15 units in the control group. During the intervention period, the administration error rates were 7.09% (188 OE with at least one error/2653 TOE) for the experimental group and 6.23% (210 OE with at least one error/3373 TOE) for the control group (*p* = 0.192). Identified risk factors (patient age, nurses’ experience, nurses’ workload, unit exposition, and interruption) were not associated with the error rate. The main error type observed for both groups was wrong dosage-form. Most errors had no clinical impact for the patient and the interruption rates were 15.04% for the experimental group and 20.75% for the control group.

**Conclusions:**

The intervention vest had no impact on medication administration error or interruption rates. Further studies need to be performed taking into consideration the limitations of our study and other risk factors associated with other interventions, such as nurse’s training and/or a barcode system.

**Trial registration:**

The PERMIS study protocol (V2–1, 11/04/2017) was approved by institutional review boards and ethics committees (CPP Ile de France number 2016-A00211–50, CNIL 21/03/2017, CCTIRS 11/04/2016). It is registered at ClinicalTrials.gov (registration number: NCT03062852, date of first registration: 23/02/2017).

**Supplementary Information:**

The online version contains supplementary material available at 10.1186/s12912-021-00671-7.

## Background

Medication errors can occur at any step in the medication process in hospitals (prescribing, dispensing, and administration). Between 1999 and 2005, errors most often originated during the administration phase of medication use (33%) in the American MEDMARX self-reported database [1]. Administration error is defined as a deviation from the prescriber’s medication order as written on the patient’s chart [2] and concerns the nurse administering the medication to the patient. Reported administration error rates are approximately 10% using the observation technique [3, 4], which has been found to be the best available method for determining the prevalence of administration errors.

Error-provoking conditions that influence administration errors include organizational factors (high workload or the number of patients under the care of the nurse), the nurse (inexperience of the nurse or inadequate training), patient factors (polymedication), and working environment (noisy, distractions, and interruptions [5–11]) [12, 13].

Several strategies to manage administration process and reduce medication errors have been proposed, including personnel-level interventions (training, nursing education, checklists, marked quiet zones, double checking, and vests) and organizational interventions (automated drug dispensing and barcode-assisted medication administration) [14, 15], with a limited impact [16–25]. In 2011, the French Health Authority recommended the introduction of a ‘Do not interrupt’ vest during the preparation and administration round to assure the medication process [26]. However, no study has used a controlled randomized multicenter design to evaluate the impact of a vest to reduce medication errors and this recommendation has not been followed in French hospitals.

The main objective of this study was to evaluate the impact of a ‘Do not interrupt’ vest on administration error rates in four French hospitals using a randomized controlled design. Secondary objectives were to evaluate the impact of the vest on the types of errors, the potential clinical impact of errors for patients, and the interruption rates during the medication process. We hypothesized was that the vest and a poster would reduce the administration error rates.

## Methods

### Study setting

The study was conducted in 29 in-patient units of four French adult hospitals from February to July 2017: two teaching hospitals (European Georges-Pompidou and Paris Saint-Joseph) and two follow-up care and rehabilitation hospitals (Corentin Celton and Vaugirard).

The European Georges-Pompidou hospital includes 814 beds divided into 43 units and the Paris Saint-Joseph Hospital, 643 beds divided into 28 units. The Corentin Celton Hospital (501 beds, 15 units) and Vaugirard Hospital (320 beds, eight units) are part of the same hospital group as the European Georges-Pompidou Hospital (Paris West Hospitals Group) but have three separate locations. Ten units of each teaching hospital (4 medical units, 4 surgical units, and 2 critical care units) and five of each follow-up and rehabilitation hospital were included. Units were selected by the directorates for head nurses and quality and were representative of each hospital’s activity.

### Trial design

This was a multicenter, cluster, controlled, randomized study conducted in French hospitals. In each center, sessions with clinical staff (nurses, doctors, and other healthcare professionals) were organized before the beginning of observation to explain the aim of the study and its design. The coordinating center was the European Georges-Pompidou hospital. The study was carried out during three consecutive periods: 1) the preintervention period, before randomization, to take into account any improvement in the nurses’ practices during the interventional studies, notably in the control groups [27], 2) the vest-wearing period to familiarize the nurses in the experimental group, and 3) the intervention period. For the experimental group, the vest-wearing rate (number of nurses wearing the vest during the round divided by the number of nurses in the unit) was evaluated after 2 weeks of familiarization by the head nurses of the units; the intervention period began if the rate was ≥75%. Otherwise, an additional familiarization period of 1 week, including specific information about the aim of the study, was applied.

### Eligibility criteria

All nurses from selected units were invited to participate. Nurses who refused to participate and those who could have worked in several units during the study were excluded to avoid contamination bias. Medication administration during emergencies was also excluded from the study.

### Randomization and random drawing

Units were randomized at the end of the preintervention period into either the experimental or control group (standard of care) by the European Georges-Pompidou Hospital Clinical Trial Unit. Randomization lists were stratified by center and medical field (critical care, surgery, and medical field), with a 1:1 allocation, and created using the random selection function of Excel, such that a similar number of units and fields were enrolled in each group. The Additional file 1 shows the distribution of randomized units per hospital.

During an administration round, only one nurse was observed. It was thus decided to divide the units into two or four areas, corresponding to a single nurse in charge of defined patients. Each day, the area was randomly drawn for observation by the Clinical Trial Unit, independently of the observers and nurses, using the random selection function of SAS® (SAS Institute, Cary, NC, USA). Data were transmitted to the observers in paper form.

### Intervention

A ‘Do not interrupt’ vest was implemented in all units of the experimental group. A poster was placed at the entrance of these units to inform patients and relatives. It explained why nurses wore this vest and asked them not to be interrupted during the medication process unless they were urgently needed. The poster and vest were designed in accordance with the communication department of the hospital. The vest consisted of a blue reflective safety jacket. A short sentence ‘Do not interrupt, I am preparing medication’ was written on the back.

Nurses in the experimental group were encouraged to wear the vest over their work clothes during all medication rounds, whether or not they were being observed. Given the short period of the investigation, it was decided to provide one vest per nurse, in accordance with the hygiene department, except in critical care, where one vest was dedicated to each patient room. Additional vests were provided to each unit to ensure their availability during administration rounds.

No vests were provided, nor posters exposed in the unit for the control group. No information on the intervention was given.

### Procedure for data collection

#### Observation technique and training

Data were collected by direct non-disguised observation [28]. The observer watched the selected nurses from medication preparation to administration to the patient. Observation was carried out by pharmacists (*n* = 6) and members of the medication process group, including head nurses (*n* = 13), head pharmacy technicians (*n* = 2), and nursing managers (n = 2). All observers came from the Paris West Hospitals Group. They received training by senior pharmacists (SB, LL). The training was evaluated using a simulated scenario during one group session. The training inter-rater concordance of observers was 96.16%.

#### Observation schedule

In each unit, a maximum of 10 rounds was planned during the intervention period following the random draw of the area by the Clinical Trial Unit. Observations at baseline were planned for each unit before the intervention to account for the natural improvement in the nurses’ practices. Administration rounds were observed during the morning (7 am–9 am), midday (11 am-1 pm), or evening rounds (5 pm–7 pm). A maximum of four midday rounds were observed during the intervention period. Indeed, fewer medications are generally administered during the midday round than during the morning and evening rounds. During a round, the administration to all patients under the care of the nurse was observed for a maximum of 10 patients.

#### Collected data

Observations were conducted using a paper-based structured observational tool. The following data were collected: characteristics of the nurse (age, sex, and years of experience), nurse workload (number of patients under the care of each nurse), time for the round, and whether the vest was removed at the end of the round in the intervention units (intervention period). Observers recorded information concerning the drug prepared and administered: name, dose, dosage form, administration route, preparation of injectable drugs (diluent, solvent, speed of administration), number of interruptions and their type, and whether the vest was worn in the experimental groups. During the two observational periods, observers were followed during one round by one of the two senior pharmacists (SB, LL). In vivo inter-rater concordance between observers was 98.64%.

### Outcome measures

#### Primary outcome measure

The main outcome was the administration error rate, excluding wrong-time errors. Wrong-time errors were not included because this type of error is difficult to observe and has relatively little impact on the patient.

Errors were detected by the identification of discordances between medication prescriptions and observed data. The comparison was performed by senior pharmacists (SB and LL) blinded from the randomization arm. All discordances were consensually evaluated with a third pharmacist (BS).

An Opportunity for Error (OE) is defined as an ordered medication dose, administered or not, as well as an unordered dose administered to a patient. The Total Opportunities for Error (TOE) is the sum of all OE. The administration error rate was then calculated as the number of OE with one or more errors divided by the TOE and multiplied by 100. The OE was the unit of analysis.

#### Secondary outcome measures

Secondary outcomes were the types of error, rates and types of interruption, and potential clinical impact of the errors. The nurses’ experiences were also assessed.

- Medication errors were classified into the eight categories of the American Society of Health-System Pharmacists [29] by senior pharmacists (SB and LL): omission error, unauthorized drug error (dose given to the wrong patient, unordered drugs), wrong dose error, wrong dosage-form error, wrong drug-preparation error (incorrect dilution or reconstitution, mixing drugs that are physically incompatible, or inadequate product packaging), wrong administration technique error (doses administered via the wrong route, different from the route prescribed, or at the wrong rate of administration), deteriorated drug error (use of expired drugs or improperly stored drugs), and other medication error (including any drug administration errors not fitting within the predefined categories).
- The potential clinical impact of errors was individually analyzed by a multidisciplinary committee composed of two physicians (AK, PD), two nursing staff members (CG, CD), and three pharmacists (SB, LL, BS) blinded to the randomization arm. All discordances were consensually evaluated. The assessment of potential clinical impact was performed using a three-category scale: no clinical impact, serious or significant clinical impact, life-threatening impact [30].
- An interruption was defined as the halting of an ongoing task to respond to an external stimulus before completing the task. Interruptions were noted for each OE. The interruption rate was calculated as the number of OE with one or more interruptions divided by the TOE and multiplied by 100. Interruptions were classified into 12 types according to Relihan et al. [18] by observers during the round: nurse, doctor, hospital porter/paramedic, other professional, relative, other patient, lack of material, noise, phone, emergency for another patient, and other source of interruption. We regrouped this classification into three categories for the results: paramedical professional (nurse, other professional, hospital porter/paramedic), medical professional (doctor), non-health professional (relative, other patient, lack of material, noise, phone, emergencies, other). We excluded task interruption for emergencies initiated by health professionals and patients.

Nurses in the experimental group were invited to complete a structured satisfaction survey developed by Westbrook et al. [31]. The surveys evaluated participant satisfaction and opinions concerning the observation, interruptions, and vest, adapted to each participant. The response categories included five-point Likert scales and yes/no questions.

### Control of bias

Several biases, such as selection bias, contamination bias, and measurement bias, were considered when writing the protocol. The selection bias was limited by random drawing of the area for each observation round. Selection of the area was indeed independent of the observers and predefined by the Clinical Trial Unit. The allocation procedure ensured an equal number of experimental units and control units at each type of hospital (teaching hospital or follow-up care/rehabilitation hospital) to minimize variation related to the type of hospital and medical center. Contamination bias was likely prevented by excluding nurses who could have worked in several units during the study. The measurement bias associated with the observation technique was limited by training of the observers and evaluation of their concordance during the study.

The risk of the Hawthorne effect due to use of the observation technique was limited by the multiplicity of observations and continuity of the observers. As in all interventional studies, there is also a potential bias inherent to implementation of an intervention. We limited such an impact by using a controlled randomized study design with two periods of data collection. Concerning statistical analysis, specific attention was given to compliance in wearing of the vest to ensure powered intention-to-treat analyses. In the experimental group, the familiarization and information sessions ensured adhesion of the nurse and physicians to wearing of the vest and limited the spirit of mockery feared by nurses.

### Statistical analysis

Analyses were performed according to the intention-to-treat principle, using data updated on December 19, 2019. We compared the characteristics of hospitals and patients in the four subgroups (corresponding to the preintervention and intervention periods for each of the two groups), as well as task interruption rates by using the Chi-2 test or the Fisher exact test, as appropriate.

In our comparison of outcomes between the two groups, we applied a cluster-specific method that took dependence among patients, care unit, and nurse (clustering effect) into account [32], by using mixed logistic regression models that contained study group (experimental group vs control), study period (preintervention or intervention), and a term for the group-by-period interaction. The fixed effects were the study period (preintervention or intervention), group (experimental or control), and the interaction between period and group and the random effects were the unit of care, the nurse, and the patient. Odds ratios were determined with their associated 95% confidence intervals and *p*-values.

The risk factors identified in the literature were studied in univariate analysis: patient (age, which could lead to polymedication), nurse (experience), organizational factors (number of patients under care during each round, number of OE per administration round), and working environment (unit configuration named “unit exposition”, number of OE with at least one task interruption). For the unit configuration, an open area of the unit was considered to be more exposed to interruption. A descriptive analysis was performed for the potential clinical impact of errors and the satisfaction survey.

Analyses were performed using the SAS (version 9.4, SAS Institute, Cary, North Carolina) and R (version 4.0) softwares. A *p*- value < 0.05 was considered significant.

### Sample size and rational for the number of TOE

The study was designed based on the hypothesis of a decrease in the administration error rate of 45% [33]. The expected reduction was calculated to achieve an error rate of 4.1%, based on a previous study with a basal administration error rate of 7.5% [34]. Considering a 5% risk and 90% probability, 1979 TOE would be required. However, 1.86 times as many TOE had to be included due to a cluster effect, including intra-unit and intra-nurse correlations of 0.005 and 0.1, respectively [34], corresponding to 3700 TOE [35]. These TOE were transposed to rounds, assuming that 30 units with observations of five patients/round with at least three medications/patient would generate 450 TOE per round. An estimation of ten rounds per unit was expected to generate 4500 TOE.

#### Ethics

The protocol was approved by institutional review boards and ethics committees (CPP Ile-de-France N°2016-A00211–50) and registered at ClinicalTrials.gov (NCT03062852, date of first registration: 23/02/2017). The study was also presented to the medical committee, the patient commission, the commission for Health, Safety and Working Conditions, and during nurse and physician information sessions in the units. Head nurse staff members collected the written consent of the nurses. Nurses were informed that the study was a direct observation study of medication administration and preparation tasks to study the impact of a ‘Do not interrupt’ vest on administration errors and interruptions. All participating nurses underwent a process of non-opposition and were made aware that participation was strictly voluntary. Participants could withdraw from the study at any time. If a nurse refused to be observed, another nurse who agreed to participate was followed.

All Data were analyzed anonymously. The Clinical Trial Unit was responsible for overall monitoring of the study to ensure quality and regulatory compliance.

## Results

### Description

**Fig. 1:**
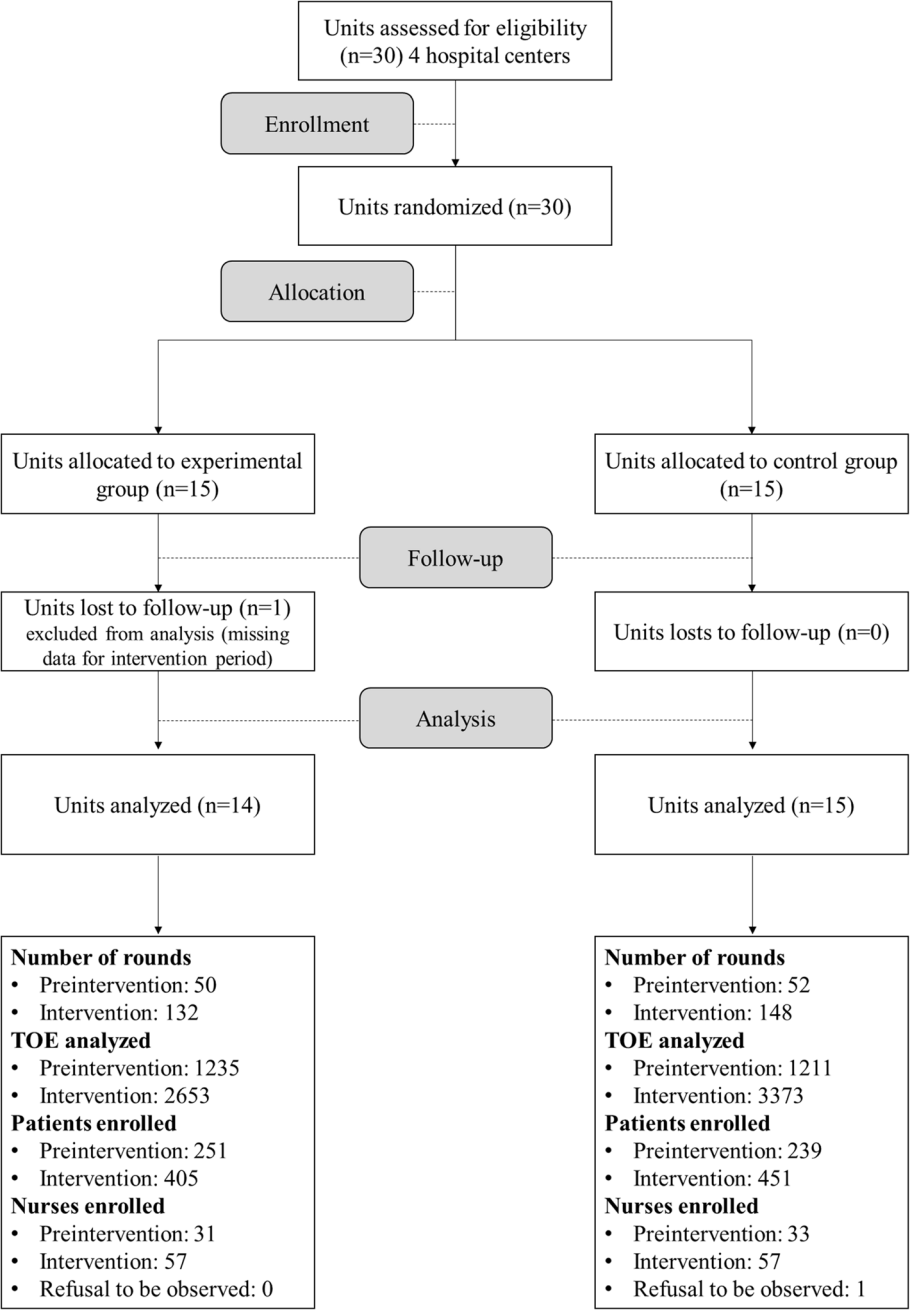
Study Flowchart

Among the 30 randomized units, one in the experimental group was excluded due to missing data for the intervention period and one nurse in the control group refused to be observed. We enrolled 178 nurses and 1346 patients during 383 medication rounds and 8472 TOE (Fig. 1). The characteristics are presented in Table 1. There were 23 trained observers. In the experimental group, the nurses largely wore the vest (90.09%, 2390 OE/2653 TOE) during the observations.

**Table 1:** Clinical characteristics for the preintervention and intervention periods, by study group

	Experimental group *n* = 14 units		Control group *n* = 15 units	
	Preintervention period	Intervention period	Preintervention period	Intervention period
**Patients (n)**	**251**	**405**	**239**	**451**
Mean age (SD), y	72.62 (18.82)	69.91 (18.58)	70.68 (19.44)	72.99 (18.18)
Male, n (%)	121 (48.21)	204 (50.37)	126 (52.72)	205 (45.45)
**Nurses (n)**	**31**	**57**	**33**	**57**
Mean age (SD), y	36.33 (10.12)	32.26 (9.86)	34.64 (9.88)	32.09 (9.78)
Male, n (%)	3 (9.68)	6 (10.53)	3 (9.09)	12 (21.05)
Mean experience (SD), y	8.96 (8.53)	6.51 (7.07)	6.53 (5.82)	7.40 (8.01)
Mean number of patients per administration round (SD)	9.68 (3.96)	8.40 (3.19)	13.13 (9.34)	11.10 (8.59)
**Total number of OE**^a^**per administration round (n)**	**1235**	**2653**	**1211**	**3373**
Morning round, n (%)	582 (47.13)	1456 (54.88)	532 (43.93)	1963 (58.20)
Lunch round, n (%)	209 (16.92)	436 (16.43)	242 (19.98)	417 (12.36)
Evening round, n (%)	444 (35.95)	761 (28.68)	437 (36.09)	993 (29.44)
**Total number of OE**^a^**per type of unit, n (%)**				
Surgical unit, n (%)	179 (14.49)	609 (22.96)	236 (19.49)	582 (17.25)
Medical unit, n (%)	1001 (81.05)	1934 (72.90)	906 (74.81)	2706 (80.23)
Critical care unit, n (%)	55 (4.45)	110 (4.15)	69 (5.70)	85 (2.52)
**Total number of OE**^a^**according to unit exposition, n (%)**				
Area exposed to interruption, n (%)	1175 (95.14)	2163 (81.53)	1003 (82.82)	2705 (80.20)
Area not exposed to interruption, n (%)	60 (4.86)	490 (18.47)	208 (17.18)	668 (19.80)

^a^*OE* Opportunity for Error

### Error rate

In total, 2446 TOE were observed during the preintervention period. The administration error rates were 4.94% (61 OE with at least one error/1235 TOE) and 6.44% (78 OE with at least one error/1211 TOE) for the experimental and control groups (Table 2).

**Table 2:** Error rates, types of errors, and potential clinical impact for the preintervention and intervention periods, by study group

	Experimental group			Control group			
TOE	Preintervention period (A) (*n* = 1235)	Intervention period (B) (*n* = 2653)	*p*-value A vs. B (*)	Preintervention period (C) (*n* = 1211)	Intervention period (D) (*n* = 3373)	*p*-value C vs. D (*)	*p*-value B vs. D (†)
**Number (%) of OE with at least one error**	61 (4.94)	188 (7.09)	0.013	78 (6.44)	210 (6.23)	0.845	0.355
**Total number of errors**^a^	**62**	**191**		**81**	**213**		
**Types of errors, n (%)**							
Wrong dosage-form error	18 (29.0)	76 (39.8)	0.170	17 (21)	84 (39.4)	0.004	0.851
Unauthorized drug error	9 (14.5)	34 (17.8)	0.686	5 (6.2)	28 (13.1)	0.138	–
Omission error	4 (6.5)	7 (3.7)	0.471	1 (1.2)	1 (0.5)	0.476	–
Wrong-dose error	30 (48.4)	67 (35.1)	0.085	47 (58.0)	79 (37.1)	0.001	0.046
Wrong administration technique error	0 (0.0)	7 (3.7)	0.199	10 (12.3)	14 (6.6)	0.169	–
Wrong drug-preparation error	1 (1.6)	0 (0)	0.245	1 (1.2)	7 (3.3)	0.452	–
**Potential clinical impact of errors, n (%)**							
No clinical impact	43 (69.4)	130 (68.1)	0.974	56 (69.1)	171 (80.3)	0.060	–
Serious or significant clinical impact ^b^	19 (30.6)	61 (31.9)		25 (30.9)	42 (19.7)		

^a^ One OE can be associated with several errors and different clinical impact

^b^ No OE had a potential life-threatening impact

* *p*-value corresponds to either a Fisher test or a Chi-2 test

† *p*-value corresponds to the interaction term of the mixed logistic regression model (see statistical methods). Due to a low numbers of OE, the model could not converge for unauthorized drug error, omission error and wrong administration technique and drug-preparation error. No calculation was possible for the *p*-value of the potential clinical impact because an OE could be associated with several errors with different clinical impact

During the intervention period (Table 2), 6026 TOE were observed. The administration error rates were 7.09% (188 OE with at least one error/2653 TOE) and 6.23% (210 OE with at least one error/3373 TOE) for the experimental and control groups (*p* = 0.355).

**Fig. 2:**
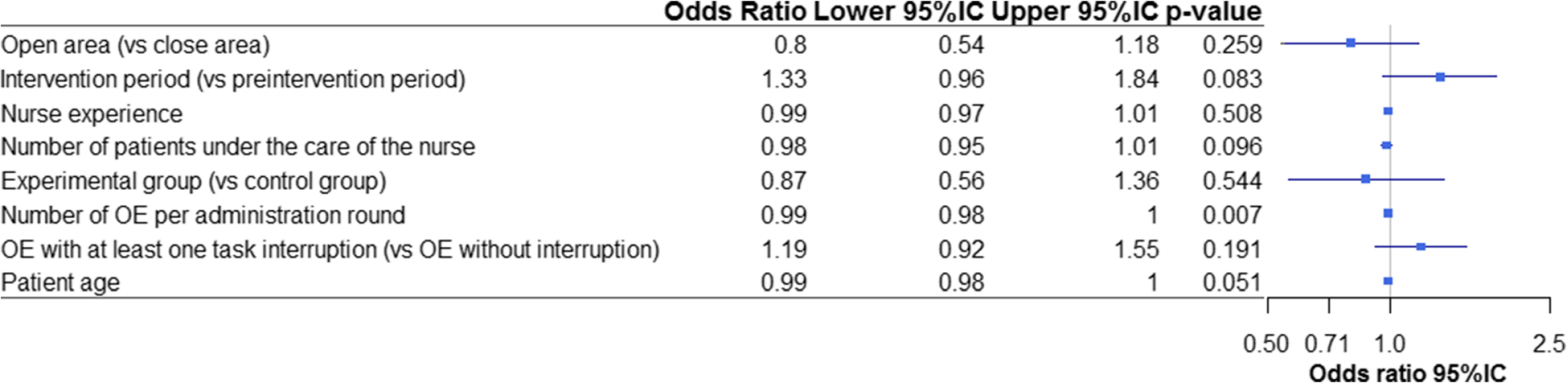
Forest plot of univariate analysis of medication errors

We did not found any risk factor associated with the error rate by univariate analysis, when considering together the two groups and the two periods (Fig. 2).

### Secondary outcomes

We observed several types of errors and interruptions for the OE during the two periods. During the intervention period, there were 188 OE with at least one error in the experimental group, resulting in 191 different errors, and 210 OE with at least one error in the control group, resulting in 213 different errors (Table 2). The main type of error observed was the wrong dosage-form during the intervention period for both groups, whereas it was the wrong dose during the preintervention period.

Most of the errors had no clinical impact for the patient (68.1 and 80.3% of the errors in the experimental and control groups, respectively) during the intervention period. There were no life-threatening errors.

During the preintervention period, the interruption rates were 13.4% (165 OE with at least one interruption/1235 TOE) and 23.78% (288 OE with at least one interruption/1211 TOE) for the experimental and control groups (Table 3). During the intervention period, the interruption rates were 15.04% (399 OE with at least one interruption/2653 TOE) for the experimental group, for a total of 479 interruptions, and 20.75% (700 OE with at least one interruption/3373 TOE) for the control group, for a total of 888 interruptions, p-value = 0.350. Most interruptions were in the non-health professional category (relative, other patient, lack of material, noise, phone, emergencies, other) for the two periods for both groups. During the intervention period, 24 administrations with at least one interruption contained at least one error in the experimental group and 52 administrations in the control group. Most of these administrations (with at least one error and one interruption) had no clinical impact.

**Table 3:** Task interruption rates for the preintervention and intervention periods, by study group

	Experimental group			Control group			
TOE	Preintervention period (A) (*n* = 1235)	Intervention period (B) (*n* = 2653)	*p*-value A vs. B (*)	Preintervention period (C) (*n* = 1211)	Intervention period (D) (*n* = 3373)	*p*-value C vs. D (*)	*p*-value B vs. D (†)
**Number of OE with at least one interruption**							
For the TOE observed, n (%)	165 (13.4)	399 (15.04)	0.182	288 (23.78)	700 (20.75)	0.031	0.350
**Total number of interruptions**^a^	204	479		364	888		
**Type of task interruptions** (at least one interruption of each type), **n (%)**	191	446		321	809		
Paramedical professional (nurse, other professional, hospital porter/paramedic)	70 (36.7)	141 (31.7)	0.252	117 (36.4)	312 (38.6)	0.790	0.002
Medical professional (doctor)	34 (17.8)	48 (10.8)	0.021	35 (10.9)	87 (10.8)	1.000	0.467
Non-health professional (relative, other patient, lack of material, noise, phone, emergencies, other)	87 (45.6)	257 (57.6)	0.007	169 (0.53)	410 (50.7)	0.600	0.593
**Potential clinical impact of errors with at least one interruption, n (%)**	**12**	**24**		**18**	**52**		
No clinical impact	8 (66.7)	17 (70.8)	1.000	12 (66.7)	39 (75.0)	0.545	–
Serious or significant clinical impact ^b^	4 (33.3)	7 (29.2)		6 (33.3)	13 (25.0)		

^a^ One OE can be associated with several interruptions

^b^ No OE had a potential life-threatening impact

* *p*-value corresponds to either a Fisher test or a Chi-2 test

† *p*-value corresponds to the interaction term of the mixed logistic regression model (see statistical methods). No calculation was possible for the p-value of the potential clinical impact because an OE could be associated with several errors with different clinical impact

In total, 29 nurses in the experimental group (51% of the nurses observed during the intervention period) completed the structured survey. The vast majority strongly agreed or agreed that interruptions occur frequently (96.6%), are a concern for patient safety (86.2%), and lead to medication errors (93.1%). The sources most often mentioned (> 50%) as being frequent sources of interruption were phone calls (72.4% of respondents), relatives (69.0%), doctors (65.5%), call buttons (62.1%), other patients (58.6%), and lack of drugs or material (58.6%). Most (62.1%) reported that being observed made no difference to them and observation made them more aware of interruptions for 48.3%. The vast majority (82.8%) reported that they wore a vest during the medication rounds and 27.6% thought that wearing the vest increased the number of times they were interrupted, whereas 37.9% thought that it had no impact. Most (62.1%) thought that wearing the vest had no impact on the time it took to administer the medications and thought that wearing a vest was not useful (55.2%).

## Discussion

In this study, the wearing of a “Do not interrupt” vest had no impact on administration error or interruption rates. We found administration error rates of 7.09% for the experimental group and 6.23% for the control group. Identified risk factors (patient age, nurse experience, nurse workload, unit configuration, and interruption) were not associated with the error rate.

Error rates were consistent with those found in intervention studies evaluating training- or technology-related interventions [14, 15, 34] and lower than those of other studies [4, 33]. Unlike our results, Verweij et al. found a significant long-term reduction in administration errors (66%) after implementing a vest in a before-and-after study between period 2 (2 weeks after implementation of the vest) and period 3 (4 months after) [36]. The most frequent errors were the absence of patient identification, wrong-time errors, and wrong reporting. These errors were not evaluated in our study. Other errors assessed in our study were rarely observed (wrong dose, wrong medication, and wrong route errors) by Verweij et al. However, these results are limited by the design of their study, which was suboptimal for evaluating the impact of intervention. Concerning interruptions, they found a 75% reduction after implementing the vest. The authors indicated that there were more factors than the vest alone that influenced the resulting effect.

Unlike other studies, we did not find an association between medication administration errors and the nurses’ experience, workload, or interruptions [12, 37, 38].

Interruption rates of approximately 50% have been reported in the literature [5, 10, 31], with nurses as the main source. Our study confirmed that interruptions occur frequently during administration, but a lower interruption rate was observed, and nurses were not the main source. This can be partially explained by the fact that double checking medications during an administration round is not recommended by the French Health Authority in adult hospitals. The only randomized controlled study available evaluated the impact of wearing a vest on the interruption rate [31]. The interruption rates in the experimental group were 56 and 38% for the preintervention and intervention periods, respectively, and in the control group, the rates reached 59 and 57%. The authors mentioned certain limitations concerning the study being monocentric, the fact that the medication error rate was not evaluated, and that nurses in the control group were aware of the vest, which could have influenced their behavior, making them more conscious of interruptions. Other small studies with a before-and-after design [18, 20, 39, 40] found a decrease in interruption rates after implementation of a vest.

The results of our survey of nurses show that being observed made them more aware of interruptions, similarly to the previous study [31]. Most thought that wearing the vest had no impact on the time it took to administer the medications and was not useful. The main sources of reported interruptions were similar to those of our study. The impact of the vest on patients was not evaluated in our study, but Palese et al. found there to be a negative impact reported by patients, inhibiting them from urgent communication with nurses [41].

Our study had several strengths. Several biases (selection bias, contamination bias, and measurement bias) were prevented due to the design. There was a risk of the Hawthorne effect due to the use of the observation technique, but it was limited by the cluster-controlled randomized design, with two periods of data collection and continuity of the observers (nurses got used to the same observer for all observations). The preintervention period was performed to evaluate the improvement of nurses’ practices without the effect of the intervention. Concerning the statistical analysis, specific attention was given to compliance in wearing of the vest to ensure powered intention-to-treat analysis.

Our study also had several limitations. We excluded one unit due to missing data for the intervention period. The intervention consisted of the vest wearing and the posters in the units but nurses were not instructed to respond in a certain manner when they were interrupted to limit the effect of the interruption [42]. This point needs to be addressed in other studies. We observed a difference in the administration error rates in the preintervention period between the experimental (4.94%) and control (6.44%) groups, indicating heterogeneity of the groups and perhaps a lack of standardization in their practice. We performed a cluster-controlled randomized study with two periods of data collection, which is adapted to intervention studies in real-life care [43]. However, a lack of comparability between studied groups is a risk of cluster clinical trials [44]. To avoid such a difference, it would probably have been better to have more homogeneous units in terms of the number of OE per administration round and the number of patients under care by each nurse. We chose to evaluate the units in terms of the specialty (medical, surgical, and critical care), with the medical units including acute and long-term units, such as rehabilitation units. Nurses in long-term units have more patients under their care, more medications to administer, and fewer prescription changes than those in acute units. In addition, patients in surgical units receive fewer medications than those in medical units. Concerning the survey, the rate of the survey response in the experimental group (51%) was higher than that in another study [31]. Finally, concerning the statistical plan, we chose the hypothesis of a decrease in the administration error rate of 45%, as observed by Poon et al. [33], who used a barcode medication administration system that prevented errors. The vest is not a barrier to administration errors like the barcode system. We believe that additional factors in the analysis of the nurses’ practices other than the vest alone would have influenced the resulting effect. Indeed, other error-provoking conditions that influence administration errors could be evaluated, such as organizational factors (low staffing, communication between health professionals, prescribing or dispensing errors, drug administration routes), individual nurse factors (fatigue, stress, unfamiliarity with certain medications), patient contributions (behavior), and problems with the supply and storage (lack of drug stocks on the wards) of medications [12].

## Conclusions

We did not demonstrate an impact of the vest in reducing administration errors. To improve further research in this area, other approaches, including personnel-level interventions (nursing education or behavioral strategies to manage interruptions) and/or organizational interventions (automated drug dispensing and barcode-assisted medication administration) could be studied.

## Supplementary Information

**Additional file 1.**

## Data Availability

All data generated or analyzed during this study are included in this published article.

## Abbreviations

CONSORT: Consolidated Standards of Reporting Trials
OE: Opportunity for Error
TOE: Total Opportunities for Errors
