# Supplementary material for: Effectiveness of a ‘do not interrupt’ vest intervention to reduce medication errors during medication administration: a multicenter cluster randomized controlled trial
Source: BMC Nurs. 2021 Aug 24;20:153. doi: 10.1186/s12912-021-00671-7 (PMC8383384; doi:10.1186/s12912-021-00671-7)
Supplement: Supplementary file 1 — Additional file 1. [file 12912_2021_671_MOESM1_ESM.docx]

**Supplemental appendix**

**Randomization list of units for each hospital**

| Hospital | Type of unit | Randomization |
| --- | --- | --- |
| European Georges-Pompidou | medical | experimental |
| European Georges-Pompidou | critical care | control |
| European Georges-Pompidou | surgical | experimental |
| European Georges-Pompidou | medical | control |
| European Georges-Pompidou | medical | experimental |
| European Georges-Pompidou | surgical | control |
| European Georges-Pompidou | surgical | control |
| European Georges-Pompidou | medical | control |
| European Georges-Pompidou | surgical | experimental |
| European Georges-Pompidou | critical care | experimental |
| Saint-Joseph | medical | experimental |
| Saint-Joseph | critical care | experimental |
| Saint-Joseph | medical | control |
| Saint-Joseph | medical | control |
| Saint-Joseph | surgical | control |
| Saint-Joseph | surgical | control |
| Saint-Joseph | medical | experimental |
| Saint-Joseph | surgical | experimental |
| Saint-Joseph | surgical | experimental |
| Saint-Joseph | critical care | control |
| Corentin Celton | medical | control |
| Corentin Celton | medical | control |
| Corentin Celton | medical | experimental |
| Corentin Celton | medical | experimental |
| Corentin Celton | medical | control |
| Vaugirard | medical | experimental |
| Vaugirard | medical | experimental |
| Vaugirard | medical | control |
| Vaugirard | medical | control |
| Vaugirard | medical | experimental |
